# Hacking Intraosseous Infusion Skills Training With 3D Printing: maxSIMIO Drilling System

**DOI:** 10.7759/cureus.31272

**Published:** 2022-11-08

**Authors:** Krystina M Clarke, Julia Micallef, Amanpreet K Jolly, Mithusa Sivanathan, Samyah Siraj, Dale Button, Christopher Patey, Adam Dubrowski

**Affiliations:** 1 Health Sciences, Ontario Tech University, Oshawa, CAN; 2 Biological Sciences, Ontario Tech University, Oshawa, CAN; 3 Paramedicine, Durham College, Oshawa, CAN; 4 Emergency Medicine, Carbonear General Hospital, Carbonear, CAN

**Keywords:** simulator development, simulation-based education, intraosseous infusion, 3d printing, healthcare simulation

## Abstract

Intraosseous (IO) infusion is an alternative way to access the vascular system to administer drugs and fluids, which is particularly helpful when the commonly used peripheral intravenous route is inaccessible. The IO procedure can be done using a drill that involves disinfecting the area, landmarking the insertion point, seating the needle in a firm and stable position in the bone, and then delivering a smooth fluid flush. However, in the current medical training landscape, access to commercially available IO drills such as the Arrow® EZ-IO® Power Driver (EZ-IO; Teleflex, Morrisville, North Carolina, United States) is difficult, especially for rural and remote areas, due to the high costs. Furthermore, the EZ-IO is not rechargeable and does not clearly indicate the remaining battery life, which could potentially put patients at risk during the IO procedure. This technical report aims to address these concerns by describing the development of an alternative, affordable, and reliable IO drilling system for training use: the maxSIMIO Drilling System. This system consists of a cordless and rechargeable IKEA screwdriver which connects to a conventional, hexagon-shaped 3D-printed drill bit needle adapter. Two needle adapters were created: Version A was designed to use a friction-based mechanism to couple the screwdriver with the EZ-IO training needle, while Version B relies on a magnetic mechanism. The major differences between the EZ-IO and the screwdriver are that a) the EZ-IO has only one rotation to advance the cannula while the screwdriver features both directions, b) the EZ-IO is not rechargeable while the screwdriver is, and c) the EZ-IO has a custom needle holder that can fit any EZ-IO training needle size while the screwdriver needs to have a custom needle adapter made to connect to the EZ-IO training needle. Overall, through this exploration, the features of the maxSIMIO Drilling System in comparison to the EZ-IO appear more accessible for IO training. Future considerations for this development include gathering clinical expertise through rigorous testing of this novel system.

## Introduction

In a centralized model of simulation-based education (Ce-SBE), trainees practice skills in simulation laboratories [1]. The COVID-19 pandemic challenged the Ce-SBE as access to simulation laboratories became limited due to physical distancing [2]. Furthermore, in rural and remote areas, a shortage of training equipment (i.e., simulators) and human capacity (i.e., medical educators) were identified as the two main contributors to the shortage of local training courses that aim to develop and maintain clinical skills [3]. In efforts to resolve these concerns, a decentralized model of simulation-based education (De-SBE) in which trainees practice skills outside of these laboratories was developed and tested during the COVID-19 pandemic [2]. This De-SBE model may hold significant implications on how simulation is delivered in the post-pandemic era as well [2]. 

Intraosseous (IO) infusion access is an alternative path to vascular access when peripheral IV access is compromised. This allows drugs, and fluids to enter circulation fast and in the same concentration as those administered intravenously [4-6]. The IO infusion skills can be performed manually using a needle or using a specifically designed drill, such as Arrow® EZ-IO® Power Driver (Teleflex, Morrisville, North Carolina, United States), referred to as EZ-IO going forward [7,8]. Generally, the process of drilling for IO infusion encompasses landmarking for the insertion point, firm, stabilized needle placement in the bone, and smooth delivery of a fluid flush [5]. Despite its potentially life-saving benefits in trauma patients, IO infusion remains an underutilized technique for obtaining vascular access in adults [9]. Commercially available IO simulators are often expensive and are not customizable to the needs of the learner [4]. Alternative do-it-yourself (DIY) IO simulators, such as animal bones, lack human anatomical features necessary for optimal learning and pose logistical and ethical concerns related to practicing on animals [6]. To resolve these concerns, additive manufacturing (AM) techniques using three-dimensional (3D) printing and silicone allow for the creation of cost-effective and anatomically realistic simulators for practicing IO infusion in situations where existing IO simulators are difficult to access [6,10,11]. 

Previously, the development and validation steps of several 3D-printed and silicone-based IO simulators that can be used for this type of training have been described [6]. These IO simulators differ in design, cost, and complexity. The commonalities they share are that they are inexpensive and easy to reproduce in rural and remote locations, provided that there is access to 3D printing infrastructure (e.g., local library or high school).

However, access to IO simulators is not the only gap impeding learning this skill. When considering drilling as a method for IO infusion, the availability of drilling equipment poses a similar barrier. For example, EZ-IO which is routinely used for clinical and training purposes [7,8] costs $399 CAD, is not rechargeable, and fails to provide information on battery life, which necessitates the purchase of a new unit once the battery runs out. Additionally, its useful life is approximately 500 insertions according to its user manual (could be less depending on actual usage, storage, and testing frequency), and does not indicate the number of uses remaining. The EZ-IO lifetime is cut short by the need to discard and repurchase it every time the battery dies, which contributes to unnecessary waste production and harmful environmental impacts - both of which could be avoided. The major concern with the EZ-IO is that it may unpredictably fail to function as the battery life is not measurable, thereby posing a potential risk in emergent situations. 

The aim of this report is to provide a description and access to free online resources for an alternative IO infusion system, the maxSIMIO Drilling System, that can be used for simulation-based training purposes. The maxSIMIO Drilling System consists of a commercially available, cordless and rechargeable IKEA screwdriver (henceforth referred to as screwdriver), and a conventional, hexagon-shaped 3D-printed drill bit needle adapter (hereafter referred to as needle adapter).

## Technical report

Context

The maxSIMIO Drilling System described in this report has been developed, produced, and used in the simulated training of advanced care paramedics and rural and remote physicians. Four collaborative groups with complementary areas of expertise collaborated to design this simulator: 1) technical designers and graduate students from maxSIMhealth laboratory, a research laboratory located at Ontario Tech University in Oshawa, Ontario, Canada (hereafter referred to as the development team); 2) clinicians from the Carbonear Institute for Rural Reach and Innovation by the Sea (CIRRIS), 3) a group of practicing rural and remote physicians who are members of the Society of Rural Physicians of Canada; and 4) advanced care paramedics from the Durham Region Health Department, Region of Durham Paramedic Services, The Central East Prehospital Care Program, Lakeridge Health, and Durham College (hereafter referred to as the clinical team). The clinician group from CIRRIS created the original design of the needle adapter using computer-assisted design (CAD) software based on market and design research [6]. The creators advise against using the system in non-simulation activities as it is not intended for clinical purposes.

Development process and the final product

**Figure 1:**
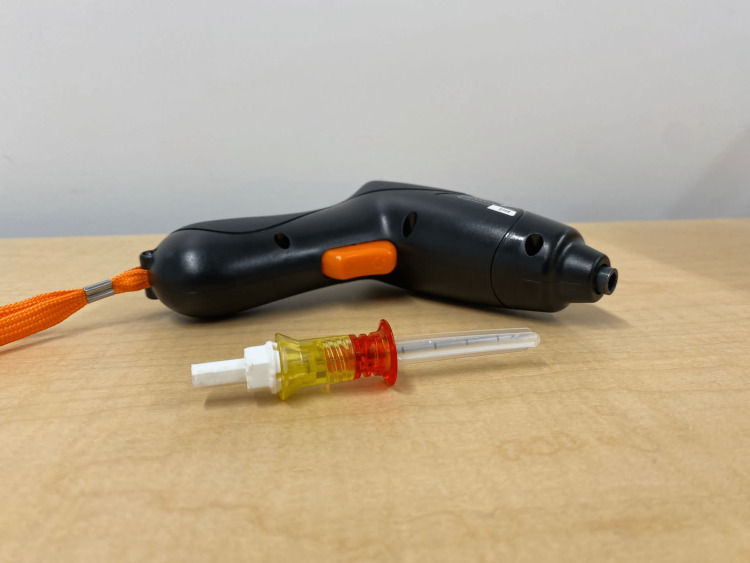
IKEA screwdriver and 15 mm Arrow® EZ-IO® Power Driver (Teleflex, Morrisville, North Carolina, United States) training needle with Version A needle adapter attached.

The maxSIMIO Drilling System consists of two components: the screwdriver and the needle adapter (Figure 1).

Screwdriver

The screwdriver is available for online or in-person purchase at IKEA (https://www.ikea.com/ca/en/p/fixa-screwdriver-lithium-ion-60196103). It features an on-off switch, which also works as a switch for clockwise/counterclockwise rotation. It has a built-in lithium-ion battery with a low self-discharge rate. The battery can be recharged using the provided power adapter, which plugs into conventional North American household power outlets. The Electronic Cell Protection prevents damage to the battery from deep discharge, overloading, and overheating. 

Needle Adapter Version A

**Figure 2:**
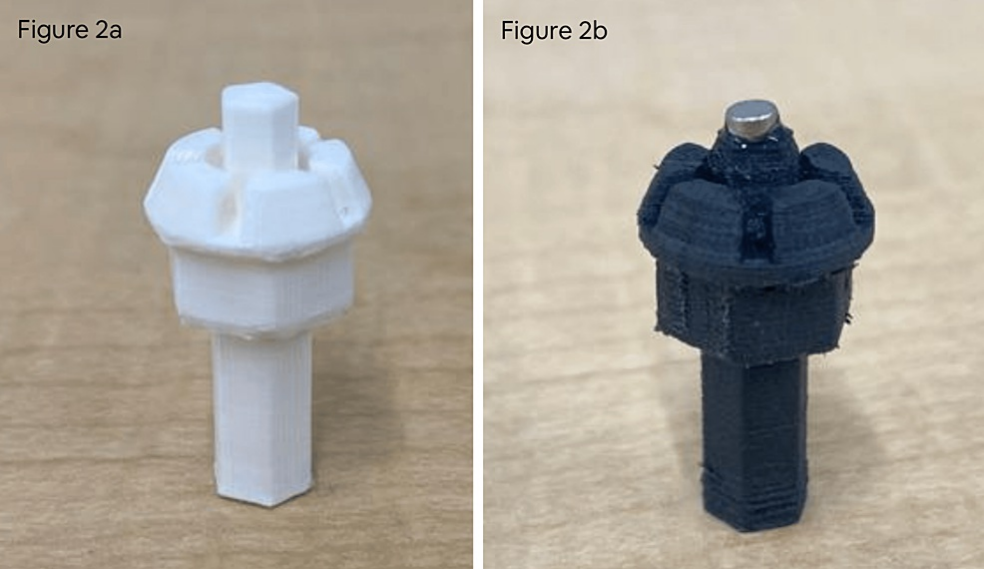
a: Photograph of Version A needle adapter (left). b: Photograph of Version B needle adapter (right).

This version was 3D-printed using BASF Ultrafuse ® (BASF SE, Ludwigshafen, Germany) polylactic acid (PLA) on Ultimaker S5 3D printer (Ultimaker B.V., Utrecht, Netherlands). The unique feature of this version of the needle adapter is that the PLA material is very flexible, and therefore it secures the EZ-IO training needle in place using a friction-based mechanism. Specifically, as illustrated in Figure 2a, the openings on the needle adapter fit snugly with the grooves on the EZ-IO training needle. However, because of the snug fit, the clinical team’s feedback was that it is difficult to remove the needle from the needle adapter after the needle is inserted into the bone. In addition, because of the physical properties of the PLA material, the finer components (i.e., grooves and tip) of the needle adapter were prone to breaking off and less precise, which resulted in even more difficulty in removal.

Needle Adapter Version B

Consequently, we have developed an alternative version of the needle adapter (Figure 2b). This version was printed using Markforged Onyx Pro (Markforged, Watertown, Massachusetts, United States) 3D printer with micro-carbon fiber-filled nylon 3D printing filament, called the Markforged 800cc Onyx Filament Spool (Markforged, Watertown, Massachusetts, United States). This ensured that the needle adapter’s finer components were more durable and precise. However, this added rigidity to the needle adapter, and therefore the friction-based coupling mechanism between the needle adapter and the training needle was not functioning. Because the EZ-IO training needles have small magnets built-in at the coupling site, the stem (Figure 2b) was reduced by 2 millimeters (mm), and a 3mm by 2mm round, multi-purpose magnet was adhered to the end of each stem using Krazy Glue (Aron Alpha®, West Jefferson, Ohio, United States) (https://www.amazon.ca/gp/product/B07FYC8H7X/ref=ppx_yo_dt_b_asin_title_o01_s00?ie=UTF8&psc=1). The designs for both versions of the needle adapter are available at https://github.com/maxSIMhealth/maxSIMIOdrill. The dimensions and costs of the needle adapter are shown in Table 1.

**Table 1:** Cost breakdown and dimensions of the needle adapters *cost does not include one 3x2mm round magnet (0.07 CAD) Markforged 800cc Onyx Filament Spool (Markforged, Watertown, Massachusetts, United States) PLA = polylactic acid

Needle Adapter Versions	X Dimension (mm)	Y Dimension (mm)	Z Dimension (mm)	Material	Amount of Material Used (g)	Cost ($CAD)
Version A	14.0	14.0	27.8	PLA	2	0.10
Version B	14.0	14.0	25.8	Markforged 800cc Onyx Filament Spool	1.69	0.52*

## Discussion

In this report, the development of an alternative IO infusion system, the maxSIMIO Drilling System, is described. The system utilizes inexpensive, commercially available power screwdrivers and a custom-developed needle adapter. By working with advanced care paramedics and rural and remote physicians, the development team has identified a screwdriver that “feels” and functions similarly to the EZ-IO, but costs only $15.00 CAD. The ergonomics of the selected screwdriver is very similar to the EZ-IO; however, the weight of the screwdriver is 356 g, whereas the EZ-IO is 455 g which constitutes efficient portability given the reduced size. The major differences between these two types of equipment are a) the EZ-IO has only one rotation to advance the cannula, while the screwdriver features both directions, b) the EZ-IO is not rechargeable, while the screwdriver is, and c) the EZ-IO has a custom needle holder that can fit any EZ-IO training needle (i.e., 15 mm, 25 mm, and 45 mm) (https://www.teleflexvascular.com/products/9058?taxon_id=27), while the screwdriver needs to have a custom needle adapter made to connect to the EZ-IO training needle, such as the one presented in this tech report. We speculate that any handheld screwdriver or small drill with a similar size, weight, and speed of rotation may be an adequate substitute for the EZ-IO. It is important to note that the counterclockwise rotation feature of the drill is not used in clinical practice (as it would pull out the needle and the remanent hole cannot be reused to secure the IO equipment to the body), and thus learners should be made aware or not allowed to use this functionality to prevent the transfer of training to real life [12]. However, in order to connect the alternative solution to the EZ-IO training needle, a custom needle adapter needs to be created.

We have created two versions of the needle adapter, both available on GitHub, licensed under CC-4.0 license for free of charge downloads (Link: https://github.com/maxSIMhealth/maxSIMIOdrill). The two versions ensure a balance between the printing materials and the utility of the needle adapter. Version A is designed to use a friction-based mechanism to couple the screwdriver with the EZ-IO training needle, while Version B relies on a magnetic mechanism. When selecting which version to use, users should consider the supply of materials available as Version A provides decreased precision due to its greater fragility. This means the Version A adapter will likely require replacement once it is disassembled from the EZ-IO training needle. Contrarily, Version B provides more precision and durability due to the micro-carbon fiber-filled nylon composition; however, this limits the functioning of the friction-based mechanism.

The production of the needle adapter of the maxSIMIO Drilling System takes approximately 20 minutes of 3D printer time through which the EZ-IO drill may be efficiently substituted at a low cost. Although future work will focus on expert-based validation and testing of the maxSIMIO Drilling System, we anticipate that it will function well to complement the IO simulators [11] in the De-SBE model of training. This will make it accessible and adaptable to many training contexts that exceed the initial pandemic-related constraints, not short of serving as backup technology in rural Emergency Departments, remote military sites, or travel.

## Conclusions

The maxSIMIO Drilling System described in this technical report is an inexpensive, modular, and extremely adaptable system that can be used to increase simulation training equipment availability in many training contexts. Coupled with IO simulators developed using similar AM techniques as described in the development team’s previous reports, this solution is poised to reduce the economic barriers to training clinical skills, such as IO insertion in both urban and rural and remote contexts. Since the lifespan of our proposed solution demonstrates improvements in the existing IO infusion technique market (e.g. EZ-IO drill discarded once the battery dies), there is an added beneficial layer of environmental consciousness that will help reduce our carbon footprint. If nurtured with the appropriate resources and environment, it is possible for the maxSIMIO Drilling System design to be translated into a final version that is safe for and capable of implementation beyond simulation-based learning scenarios and into clinical, real-world settings.
